# A margin for error in grasping: hand pre-shaping takes into account task-dependent changes in the probability of errors

**DOI:** 10.1007/s00221-019-05489-z

**Published:** 2019-02-12

**Authors:** Bruce D. Keefe, Pierre-Arthur Suray, Simon J. Watt

**Affiliations:** School of Psychology, Bangor University, Penrallt Rd., Bangor, Gwynedd LL57 2AS UK

**Keywords:** Grasping, Visuo-motor control, Visual uncertainty, Margin for error, Grip aperture

## Abstract

Ideal grasping movements should maintain an appropriate probability of success, while controlling movement-related costs, in the presence of varying visual (and motor) uncertainty. It is often assumed that the probability of errors is managed by adjusting a margin for error in hand opening (e.g., opening the hand wider with increased visual uncertainty). This idea is intuitive, but non-trivial. It implies not only that the brain can estimate the amount of uncertainty, but also that it can compute how different possible alterations to the movement will affect the probability of errors—which we term the ‘probability landscape’. Previous work suggests the amount of uncertainty is factored into grasping movements. Our aim was to determine whether grasping movements are also sensitive to the probability landscape. Subjects completed three different grasping tasks, with naturally different probability landscapes, such that appropriate margin-for-error responses to increased uncertainty were qualitatively different (opening the hand wider, the same amount, or less wide). We increased visual uncertainty by blurring vision, and by covering one eye. Movements were performed without visual feedback to isolate uncertainty in the brain’s initial estimate of object properties. Changes to hand opening in response to increased visual uncertainty closely resembled those predicted by the margin-for-error account, suggesting that grasping is sensitive to the probability landscape associated with different tasks. Our findings therefore support the intuitive idea that grasping movements employ a true margin-for-error mechanism, which exerts active control over the probability of errors across changing circumstances.

## Introduction

The elegant efficiency of natural grasping movements is evident in how the posture of the hand is shaped ‘in-flight’ to anticipate the properties of target objects (Jeannerod 1984, 1988; Marteniuk et al. 1990). This hand pre-shaping is often thought to include a margin for error, designed to prevent mistakes such as knocking into objects, or failing to grasp them altogether, in face of visual (and motor) noise (Jakobson and Goodale 1991; Winges et al. 2003; Schlicht and Schrater 2007a; Christopolous and; Schrater 2009; Takemura et al. 2015). The idea that the grasping system responds adaptively to visual uncertainty emerges from the observation that increased uncertainty typically results in wider opening of the hand (Wing et al. 1986; Jakobson and Goodale 1991; Schlicht and Schrater 2007a). At face value, this behaviour resembles a “strategy” of erring on the side of caution, acting to mitigate the otherwise increased probability of error (Hibbard and Bradshaw 2003; Melmoth and Grant 2006). Although the margin-for-error account is intuitive, it implies non-trivial underlying processes. Specifically, it suggests that the probability of grasping errors is actively controlled, presumably to some criterion level, over varying conditions. We argue that this requires not only access to the precise amount of visual uncertainty, but also knowledge of how different possible movements increase or decrease the probability of errors. We refer to the latter as the ‘probability landscape’ associated with the task. Evidence that the precise magnitude of visual uncertainty is factored into grasp programmes comes from the finding that there is a systematic relationship between the degree of visual uncertainty and grasp opening (Schlicht and Schrater 2007a). In this experiment we consider whether grasp programming also factors in the probability landscape. Specifically, we explored whether the responses to increased visual uncertainty were appropriate across tasks with qualitatively different probability landscapes. We reasoned that such sensitivity would provide compelling evidence for active control of the probability of errors, as the margin-for-error account implies.

Movements such as grasping can be characterised as optimisation problems, over a wide range of variables (e.g. Flash and Hogan 1985; Harris and Wolpert 1998; Todorov and Jordan 2002). An ideal movement has a high probability of success, while managing other costs such as energy expenditure, movement time, comfort etc. The value of success and failure also matters. Knocking over a wooden block in a visuo-motor control laboratory has different consequences than spilling hot coffee on one’s hand, for instance, and it can be worth expending greater energy for greater ‘reward’ (Shadmehr et al. 2016). The process of determining how grasping can best be achieved in a given situation can therefore be construed as minimising an overall cost function that considers intrinsic movement costs, the probability of different outcomes, and the value of those outcomes (Trommershäuser et al. 2008; Todorov and Jordan 2002; Wolpert and Landy 2012). A fundamental premise is that this process is subject to noise (uncertainty), rendering it probabilistic in nature (Trommershäuser et al. 2008). At the ‘front end’, visual estimates of object and scene properties, and feedback, are subject to noise. Noise is presumably also introduced at various intermediate neural processing stages, such as co-ordinate transformations. Then motor noise contributes to variability in motor output per se (Trommershäuser et al. 2003; Schlicht and Schrater 2007b; Takemura et al. 2015).

As noted above, one source of noise is the brain’s estimates of the spatial properties of the object to be grasped. Grasp kinematics reveal that object size, location, etc. are estimated from vision prior to movement onset, allowing the grasping hand to be pre-shaped so as to anticipate the final posture required (Jeannerod 1984, 1988; Marteniuk et al. 1990; Jakobson and Goodale 1991). Hand opening, for instance, reaches a clearly identifiable peak (maximum grip aperture) partway through the movement, which, although wider than the object, varies highly reliably as a function of object size. Estimates of object properties are necessarily noisy, however, and the amount of noise or uncertainty is not fixed, but varies substantially, even in normal viewing. For example, geometrical factors such as the distance and orientation of object surfaces, their visible texture, and content of the surrounding scene all result in variations in uncertainty (e.g., Gepshtein and Banks 2003; Knill and Saunders 2003; Hillis et al. 2004; Keefe et al. 2011). An ideal movement programme should take account of these changes because they have implications for the probability of success. Consider the effects of increased visual uncertainty. This increases the range of possible locations of a target object’s surfaces (Schlicht and Schrater 2007a), corresponding to an increased range of possible sizes/positions the object could have. Thus, if increased uncertainty simply propagates to more variable movement programmes, the probability of failing to enclose the object with the hand increases, and so the probability of errors (here, the digits colliding with the object) increases.

According to the margin-for-error account, the grasping system mitigates this increased probability of errors by adjusting the bias in the programmed hand opening. In the above example it is intuitive that increasing hand opening will reduce the probability of errors: the grasp cannot be opened too wide to enclose the object, but can be opened not-wide-enough [see Christopolous and Schrater (2009), for a more complex case]. Yet, the underlying computations required to achieve precise control over the probability of errors (as opposed to a simple heuristic response) are non-trivial. Two related pieces of information are required. First, the magnitude of uncertainty in the estimate of object properties must be known precisely (i.e., the probability distribution describing the range of possible locations of the object’s surfaces). Second, the system must know how different possible movements affect the probability of success or failure, which we refer to as the probability landscape associated with the task. As noted above, for normal grasping movements the probability landscape is asymmetrical with respect to hand opening, in that increasing it decreases the chance of errors and vice versa, but other tasks have different probability landscapes associated with them. In principle, combining these two pieces of information can then yield the required alteration to grasp opening to control the probability of errors to the desired level, given current circumstances.

Definitive evidence that the grasping system is sensitive to the first of these pieces of information—the degree of visual uncertainty—is (surprisingly) sparse. Many studies have manipulated the ‘quality’ of visual information available. However, because these studies typically employed binary manipulations, comparing normal vs. degraded vision (binocular vs. monocular vision, for example), it is not possible to determine unambiguously whether changes to grip apertures reflected sensitivity to the precise magnitude of visual uncertainty, or a kind of heuristic strategy, or ‘ballpark’ response (e.g. Servos et al. 1992; Jackson et al. 1997; Watt and Bradshaw 2000; Loftus et al. 2004; Melmoth and Grant 2006; Keefe et al. 2011). One exception is a study by Schlicht and Schrater (2007a), who manipulated visual uncertainty more parametrically, by varying the retinal eccentricity of the object and moving hand (subjects fixated an eccentric ‘target’ positioned at 0°–80° eccentricity, in 10° steps). They found a highly reliable relationship between visual eccentricity and hand opening, with increasingly eccentric (increasingly uncertain) visual information resulting in systematically larger grip apertures, demonstrating sensitivity to the amount of visual uncertainty.

**Fig. 1:**
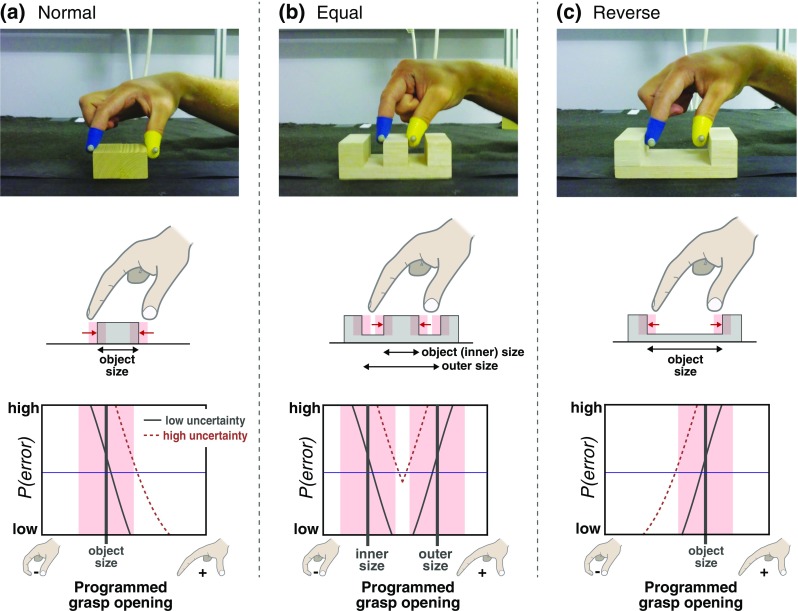
The three tasks and associated probability-landscape conditions. **a** The normal probability condition. **b** The equal probability condition. **c** The reverse probability condition. The top row shows each object type being grasped. Rubber thimbles worn on the finger and thumb, and the motion capture markers, are also visible (see “Methods”). The middle row depicts increased visual uncertainty in cartoon form. The images show side views of objects in each condition. The arrows below the objects indicate the how we defined object size in each case (in the equal condition, object size = inner size). The arrows pointing at the object surfaces depict the direction of prototypical final movements of the digits onto the object surfaces. The shaded zones (pink, in colour versions) depict the range of possible locations of the objects’ surfaces, given an arbitrary amount of visual uncertainty (in reality this is continuous rather than discrete, reflecting an underlying probability distribution). The bottom row depicts, schematically, hypothetical probabilities of grasping errors as a function of the programmed grasp opening for each task. For simplicity, we depict uncertainty in terms of its effect on the singular value object size, but in reality it corresponds to uncertainty in the location of the object’s surfaces, and so can also be thought of as positional uncertainty. The vertical line(s) show an example object size, and the horizontal lines show an arbitrary criterion level for the probability of errors. The pink shaded zones show the increased range of possible sizes (locations of the object’s surfaces) with high uncertainty, as previously. The solid curves depict how the probability of errors varies with hand opening for low visual uncertainty (assuming some fixed level of motor noise). The dashed curves depict the same relationship for high visual uncertainty

In this experiment we examined whether grasp pre-shaping is sensitive to the other component of the margin-for-error account: the probability landscape associated with the task. The above studies examined similar movements (precision grasping, in which the index finger and thumb close onto the outer surfaces of a simple object) for which the appropriate response to increased uncertainty was qualitatively the same (increased grasp opening). We exploited naturally occurring changes in the qualitative shape of the probability landscape that result from different tasks. The three tasks used in our experiment are explored in Fig. 1. The left column (Fig. 1a) depicts the normal probability condition, in which subjects grasped in the normal manner, closing the finger and thumb on the outer surfaces of the objects. Here the probability landscape is asymmetrical with respect to grip aperture, as described above. All else being equal, the wider the hand is opened the lower the probability of failing to grasp the object, so the probability of errors falls monotonically as the programmed grasp opening increases (solid line in the bottom panel). As visual uncertainty increases, the range of possible locations of the object surfaces increases (pink shaded zones in the middle and bottom panels). Now, for the same hand opening, the probability of errors increases because the object will fall outside the hand opening on a greater proportion of occasions (dashed line in the bottom panel). The criterion error level can be met, however, by programming a wider hand opening. Thus, the appropriate response to increased uncertainty is to increase grip apertures. The middle column (Fig. 1b) depicts the equal probability condition, in which subjects grasped objects by inserting the digits into two slots to grasp a central bar. Because it is possible to collide with both the inner and outer surfaces of the slots, opening the hand wider or less wide will result in approximately equal increases in the probability of colliding with the upper surface of the object (the probability landscape is symmetrical with respect to hand opening). In this case, increased uncertainty results in a narrower ‘zone’ into which the digits must be inserted to achieve the criterion error level, and changing the bias of the programmed grasp opening therefore does not provide effective control over the probability of errors. Thus, the margin-for-error account predicts no change in grasp opening in this situation, because there is no adjustment to grip aperture that can mitigate the otherwise increased probability of errors. (Note, with sufficient uncertainty it may be impossible to achieve the criterion error level with any programmed grasp opening.) The right column (Fig. 1c) depicts the reverse probability condition, in which a u-shaped object was grasped by moving the digits outwards to touch its inner surfaces. Here, the converse pattern to normal grasping applies. Hand opening can be too large, but not too small, and so the probability landscape is again asymmetrical, but reversed with respect to normal grasping, and the criterion error level can be met by programming a smaller hand opening. In this condition, sensitivity to the probability landscape would therefore be indicated by decreased grip apertures in response to increased uncertainty—the opposite of normal grasping. If, instead, the grasping system is insensitive to changes in the probability landscape, grip apertures would be expected either to increase in response to uncertainty in all three conditions, or for no clear pattern to emerge in the unusual situations of the equal and reverse conditions. We increased visual uncertainty in two different ways—by blurring vision, and by removing binocular information—reasoning that this may provide converging evidence. In all conditions, we prevented vision of the target and hand following movement onset so that any effects were attributable to changes in uncertainty in initial estimates of object properties used to plan movements (our experimental question), and not to differences in the quality of visual feedback, which may have varied across the different tasks.

## Methods

### Subjects

Fourteen subjects took part in the experiment (13 right-handed, one left-handed; 6 male, 8 female, aged 18–35 years old). All had normal or corrected to normal vision, including normal binocular depth perception (stereoacuity better than 40 arcsec). None reported any motor deficits. Subjects gave informed consent and were paid for their participation. The procedures were approved by the Ethics Committee of the School of Psychology, Bangor University, and were in accordance with the Declaration of Helsinki.

### Apparatus and stimuli

**Fig. 2:**
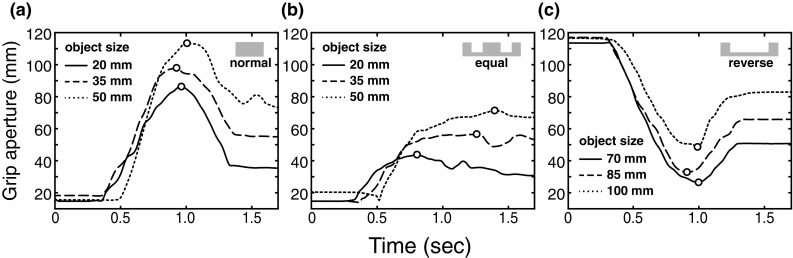
Example grip aperture profiles for each task, from one subject. **a** 3-D distance between finger and thumb markers (grip aperture) as a function of time, in the normal condition. The three curves depict three individual trials, with binocular viewing, for movements to the smallest (solid line), middle-sized (dashed line), and largest (dotted line) object. The circular symbols on each curve show the points identified as maximum grip aperture in each case. **b, c** Example grip aperture profiles for the equal and reverse conditions, in the same format as **a** (except minimum grip apertures are indicated in **c**)

The three different grasping tasks, and object types, used to manipulate the probability landscape are shown in Fig. 1. Subjects were seated at a table, and positioned with their eyes ~ 400 mm above the table surface, directly above a start button aligned with their body midline. A chin rest was used to stabilise head position. In all conditions, subjects grasped objects using only the thumb and index finger of their preferred hand. In the normal and equal conditions subjects began each trial with these digits lightly pinched together, pressing the start button. In the reverse condition subjects began each movement with an open grasp, with the thumb pressing on the start button, and the index finger resting on a 10 × 10 mm pad 120 mm in front of the start button. Our intention was for the reverse condition to be an ‘inverted’ analogue of normal grasping, in which subjects first closed the hand to a size smaller than the object, and then opened it wider at the end of the movement to ‘grasp’ the object (see Fig. 2). The table surface was covered in matt black cardboard, and the scene was lit by normal fluorescent lighting. Vision of the scene was controlled using a Liquid Crystal “smart glass” panel (PolyVisionTM, United Kingdom) positioned ~ 50 mm in front of subjects’ eyes. The state of the panel—transparent or opaque—was controlled by the experiment computer, and when opaque it occluded vision of the hand and stimuli.

Subjects wore rubber thimbles (diameter approximately 15 mm) on the finger and thumb in all conditions (see Fig. 1). This was done to equate friction between the object surfaces and digits across the different tasks. Otherwise, in the reverse condition, the nails of the digits would have contacted the object surfaces (as opposed to the pulpar surfaces in the other conditions), resulting in insufficient friction to easily grasp them, which may have affected the margin-for-error response.

Each probability condition required a different set of target objects. Within each condition we used a large number of object sizes (seven). We also presented objects at pseudo-randomised distances (see below). This factor was not of experimental interest, but the combination of a large number of sizes and random distances was intended to minimise learning of object properties and/or the required movements, which has been shown to reduce the effects of degrading visual information (by providing another signal to the required movement; Keefe and Watt 2009). The size of the objects varied along the dimension along which they were grasped (Fig. 1). In the normal task, the objects had front-to-back sizes of 20, 25, 30, 35, 40, 45 and 50 mm. In the equal task, the sizes of the central blocks were the same as the sizes in the normal condition. The slots in which the digits were placed were 25 mm wide (and 22 mm tall). In the reverse task the distances between the grasped surfaces were 70, 75, 80, 85, 90, 95, and 100 mm. Object distances were generated by using six ‘base’ distances (200, 250, 300, 350, 400 and 450 mm from the start button), and then adding a random value between 0 and 50 mm (in 5 mm increments) on each trial. This had the effect of distributing the distances used in each block throughout the range 200–500 mm, with a random ‘jitter’. The objects were presented on the table surface along the subjects’ body midline.

Grasping movements were recorded using a ProReflex infrared motion capture system (Qualisys AB, Sweden). The system captured the *x, y, z* positions of markers attached to the thumb and finger thimbles (visible in the top row in Fig. 1), at 240 Hz.

There were three viewing conditions: binocular viewing, blurred binocular viewing, and monocular viewing. In the binocular condition the scene was viewed normally, with both eyes, and so visual uncertainty was at a typical level. In the blurred binocular viewing condition the scene was viewed through a translucent plastic diffusing film (made from stage-lighting gel) attached to the smart-glass panel in front the eyes, which increased visual uncertainty by blurring the scene. In the monocular viewing condition subjects wore an eye patch over their right eye, which increased visual uncertainty by eliminating binocular depth cues (ocular vergence and binocular disparity; Hillis et al. 2004; Keefe et al. 2011).

### Procedure

With the exception of the different tasks (and starting posture for the reverse condition), the procedure was the same for all conditions. On each trial, the object was presented for 2 s followed by an audible beep, which was the signal to initiate the movement. In the normal and equal conditions subjects were instructed to pick up the objects front to back using their thumb and index finger (grasping the central block in the case of the equal grasping condition; Fig. 1). In the reverse condition subjects were instructed to pick up the objects by pushing against their opposing inner surfaces using their thumb and index finger (Fig. 1). Releasing the start button extinguished the subjects’ view, so that grasps were performed without visual feedback. Reaches that were initiated before the start signal, or > 600 ms after it, were considered void (void trials were repeated at the end of the block, such that we obtained complete data sets that met the response criteria for all subjects in all conditions). The number of void trials was small overall, and did not vary systematically with viewing condition (see below). The experiment was a within-subjects design: all subjects completed all three tasks (normal, equal, and reverse) under all three viewing conditions (binocular, blurred binocular, and monocular). Trials were blocked by viewing condition and by grasping task (normal, equal, or reverse). Viewing condition was necessarily blocked for practical reasons. Grasping task was blocked to give subjects the best opportunity to learn the novel probability landscapes, and to minimise the likelihood of our study confounding appropriate margin-for-error responses per se with difficulties switching rapidly between tasks/probability landscapes. A single block consisted of each object size presented at each object distance (plus jitter), making 42 trials. Each block was repeated twice for each viewing condition/task. Subjects therefore completed 84 trials within each viewing condition and task, and 756 trials (18 blocks) in total. Within each block, trial order was randomised. The experiment was completed in two halves, each containing one block of each condition/task. Within each half, block order was randomised, with the constraint that the same task/probability-landscape condition was not completed on consecutive blocks. Each subject took around 5 h to complete the experiment, split across several sessions (completed across several days). The median number of void trials per condition/task (84 valid trials) was 5, 7 and 5 in the normal, equal and reverse probability-landscape conditions, respectively (we analysed medians because void-trial rates were bimodal rather than normally distributed: most subjects had consistently low rates, while a small number had consistently higher rates). Friedman tests showed that void-trial rate was not affected by viewing condition in any of the probability-landscape conditions (test statistic and *p* value for normal, equal and reverse conditions, respectively: *χ*^2^ = 4.82, *p* = 0.090; *χ*^2^ = 0.717, *p* = 0.70; *χ*^2^ = 0.311, *p* = 0.860).

### Predictions

Our experiment was designed to address a specific a priori question about the grasping system’s response to visual uncertainty. We therefore constrained our analyses to testing a small number of well-specified, directional predictions, for specific dependent measures, rather than carrying out global analyses. Our hypothesis relates to grasp pre-shaping, and so our primary dependent measure was maximum grip aperture (minimum grip aperture in the case of the reverse probability condition). As discussed in the Introduction (and see Fig. 1), the margin-for-error account predicts different effects of increased uncertainty in normal, equal, and reverse tasks/probability conditions (larger, unchanged, and reduced grip apertures, respectively). We did not know the magnitude of uncertainty increase due to the blur and monocular manipulations, and so we could not make comparative predictions about the two conditions, beyond noting that they should result in qualitatively similar effects.

We prevented visual feedback in all conditions to isolate effects of uncertainty in estimates of object properties per se. For normal grasping, this manipulation itself would be expected to result in larger maximum grip apertures than under natural viewing (itself thought to be a margin-for-error response to loss of visual feedback; Jakobson and Goodale 1991; Connolly and Goodale 1999; Churchill et al. 2000). Typically, effects of degrading vision on hand opening are largest at small hand openings, and reduce systematically as ‘baseline’ hand opening increases (below the biomechanical upper limit on hand opening; Keefe and Watt 2009). Thus, predicted further increases in grip aperture (due to visual uncertainty) in the normal condition are likely to be most clear when grasping the smallest objects, where there is the most ‘headroom’ for grip aperture increases (see Keefe and Watt 2009, for a similar example). We therefore evaluated statistical significance in this condition using planned pairwise comparisons (one-tailed *t* tests), comparing (i) binocular viewing vs. blurred binocular, and (ii) binocular viewing vs. monocular for the subset of grasps to the smallest object. The converse pattern is expected for the reverse probability condition. There is a limit on minimum grasp opening, which would be expected to have the greatest influence at the smallest object sizes. Thus, the effects of increased visual uncertainty should be most clearly seen when grasping the largest objects. In this condition, we therefore carried out planned pairwise comparisons between the same conditions as above, but on grasps to the largest object size. It is not meaningful to specify particular comparisons, a priori, for the equal probability condition because the prediction is for no effect of visual uncertainty on maximum grip apertures (i.e., a null result) at all object sizes. We tested these effects with the same statistical criterion (one-tailed probability, at the middle object size of 35 mm) to err on the side of finding a false-positive effect of visual uncertainty (i.e., to be conservative with respect to accepting the null hypothesis).

## Results

### Data processing and computing grip aperture data

For each trial, the 3-D co-ordinates of each marker were low-pass filtered (Butterworth filter, 24 Hz cut-off). Grip aperture profiles for each trial were computed by calculating the Euclidean distance between the thumb and finger markers on each frame. We then identified the point of inflection in each grip aperture profile that represented the largest margin for error: the maximum grip aperture for the normal and equal probability conditions, and the minimum grip aperture for the reverse condition. Figure 2 shows example grip aperture profiles from each probability condition (under binocular viewing). As expected, profiles in the normal condition followed the typical pattern (Jeannerod 1984, 1988), with a clearly identifiable maximum. In the equal condition, maximum grip aperture was less pronounced because grasp opening more closely matched the separation of the slots in the object for the latter part of the movement, but there was still a clearly identifiable maximum before contact with the object (Fig. 2b). In the reverse condition, grip aperture profiles closely resembled inverted normal profiles, with a clearly identifiable minimum grip aperture.

### Grip aperture scaling

A canonical property of normal grasping movements is that maximum grip aperture scales highly reliably with changes in object size. This indicates that size is encoded and used to programme movements that anticipate the final grasp posture required (Jeannerod 1984, 1988; Marteniuk et al. 1990). Sensitivity of movements to object size can be considered a precondition of an appropriate margin-for-error response, because it does not make sense that movements should be sensitive to the required margin for error around a given size if they are insensitive to size per se. The reverse-probability condition, in particular, required subjects to make a movement that may have been novel to them. We therefore considered the possibility that they may not have learned to scale hand opening to anticipate object size, in which case they would not be expected to show an appropriate margin-for-error response to increased uncertainty. To determine whether this was the case, we examined the slopes of the functions relating each subject’s maximum grip apertures (minimum, for the reverse condition) to object size. We did this separately for each probability condition, for grasps made with (unblurred) binocular vision, where the scaling should be greatest. We characterised the degree of grip aperture scaling for each subject as the slope of the best-fitting linear regression to their data in the relevant condition (collapsed across object distance). We used all of the individual trials as input to the regression (rather than means), allowing us to calculate 99% confidence intervals around the slope estimates. We then evaluated whether these slopes were reliably different than zero—our definition of grip aperture scaling—by determining whether the 99% confidence intervals on each slope estimate overlapped zero.

As might be expected, in the normal condition all subjects showed highly significant grip aperture scaling. The average slope of the scaling functions (grip aperture as a function of object size) was 0.74 (SEM = ± 0.039), which is slightly higher than typical for movements made without visual feedback (e.g. Jakobson and Goodale 1991; Churchill et al. 2000). A similar pattern was evident in the equal condition. All subjects again showed significant grip aperture scaling and the average slope of the scaling functions was 0.78 (SEM = ± 0.058). In the reverse condition, however, there was no significant grip aperture scaling for four of the 14 subjects (scaling-function slopes of 0.09, 0.10, 0.11, and 0.17). Based on the reasoning outlined above, these subjects would not be expected to show an appropriate margin-for-error adjustment to grip apertures, and so their data were not included in the analyses for the reverse condition (their data for the other two conditions were included). The remaining subjects showed statistically significant grip aperture scaling, indicating sensitivity to object size, albeit with a comparatively low average scaling-function slope of 0.35 (SEM = ± 0.040).

### Effects of visual uncertainty on grip aperture

**Fig. 3:**
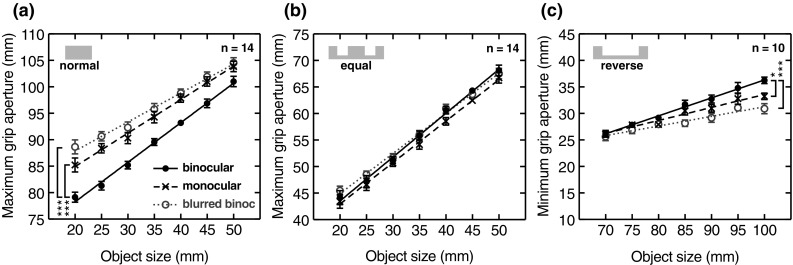
Grip aperture results. **a** Average maximum grip aperture as a function of object size in the normal probability condition. The symbols denote the three different viewing conditions. The lines are the best-fitting linear regressions to the data in each case. **b** Average maximum grip aperture for the equal probability condition, in the same format as **a. c** Average minimum grip aperture in the reverse probability condition, again in the same format as **a**. In all plots, error bars denote ± 1 SEM (between subjects). Asterisks denote statistically significant pairwise comparisons (see Table 1 for *p* values)

Figure 3a plots the overall average maximum grip apertures (*n* = 14) for normal grasping, as a function of object size (collapsed across object distance), for each viewing condition. In line with the predictions of the margin-for-error account, and with previous data, increasing uncertainty either by covering one eye (monocular viewing), or blurring vision, resulted in increased maximum grip apertures. As expected, the data show a reduced effect at large object sizes (Keefe and Watt 2009), but the effect of visual uncertainty is evident at all object sizes. As outlined in the “Predictions” section, we evaluated the statistical significance of these effects by conducting planned paired t tests (one-tailed) comparing grip apertures with (i) binocular vs. monocular vision, and (ii) binocular vs. blurred binocular vision, for grasps to the smallest object size (for which the effects were expected to be largest; Keefe and Watt 2009). The results of these tests are presented in Table 1. Because we tested a small number of planned comparisons, evaluating predictions that were different for each task, we used an alpha level of 0.05 to determine significance (i.e., uncorrected for multiple comparisons). Exact *p* values are reported in Table 1. For normal grasping, both manipulations of visual uncertainty caused statistically significant increases in maximum grip aperture. These results therefore closely resemble the typical effects interpreted as a margin-for-error response in previous reports (e.g., Jackson et al. 1997; Watt and Bradshaw 2000; Loftus et al. 2004; Melmoth and Grant 2006; Schlicht and Schrater 2007a; Keefe et al. 2011).

**Table 1:** Statistical effects of increasing visual uncertainty

Comparison	Probability-landscape condition		
	Normal (20 mm object)	Equal (35 mm object)	Reverse (100 mm object)
Binocular vs. monocular	*t*(13) = 4.29, *p* < 0.001*	*t*(13) = 1.18, *p* = 0.130	*t*(9) = 2.38, *p* = 0.021*
Binocular vs. blurred	*t*(13) = 6.00, *p* < 0.001*	*t*(13) = 0.11, *p* = 0.457	*t*(9) = 6.58, *p* < 0.001*

Asterisks denote statistically significant pairwise comparisons

*T* tests (one-tailed) between (i) binocular and monocular vision conditions, and (ii) binocular and blurred binocular vision conditions, for each probability-landscape condition

Figure 3b plots maximum grip apertures in the equal condition, in the same format as Fig. 3a. Here, the margin-for-error account predicts no effect of visual uncertainty on maximum grip apertures because both wider and narrower hand openings increase the probability of errors similarly (see Fig. 1 and surrounding discussion). This is what we observed: maximum grip apertures were very similar in all three viewing conditions. *T* tests (for grasps to the 35 mm object size; see “Predictions”) showed that neither manipulation of visual uncertainty had a statistically significant effect on grip apertures (Table 1) (note that because we had no predictions about the sign of any effects, we used the tail of the distribution that yielded the lowest *p* value, so as to be most conservative with respect to accepting the null hypothesis.). Thus, in the equal condition, too, subjects’ responses to increased visual uncertainty were consistent with the margin-for-error account.

Figure 3c plots average minimum grip apertures (*n* = 10) in the reverse condition, in otherwise the same format as Fig. 3a, b. Here, the margin-for-error account predicts smaller minimum grip apertures with increasing visual uncertainty. Overall, the results were again consistent with predictions. As expected, a floor effect is evident in the data, with no effect of visual uncertainty at the smallest object size (note that in the binocular viewing condition, where visual uncertainty is lowest, average grip aperture when grasping the smallest object—the separation between markers on the sides of the digits—was just ~ 25 mm, which is already close to the physical minimum). With larger object sizes, effects of visual uncertainty are increasingly evident. For grasps to the largest object, both monocular vision and blurred vision resulted in significantly smaller minimum grip apertures (Table 1). Overall, the results for the reverse condition also therefore closely resemble margin-for-error adjustments to grip aperture in response to increased visual uncertainty, given a different probability landscape/task.

### Grip aperture/movement speed trade-off?

**Fig. 4:**
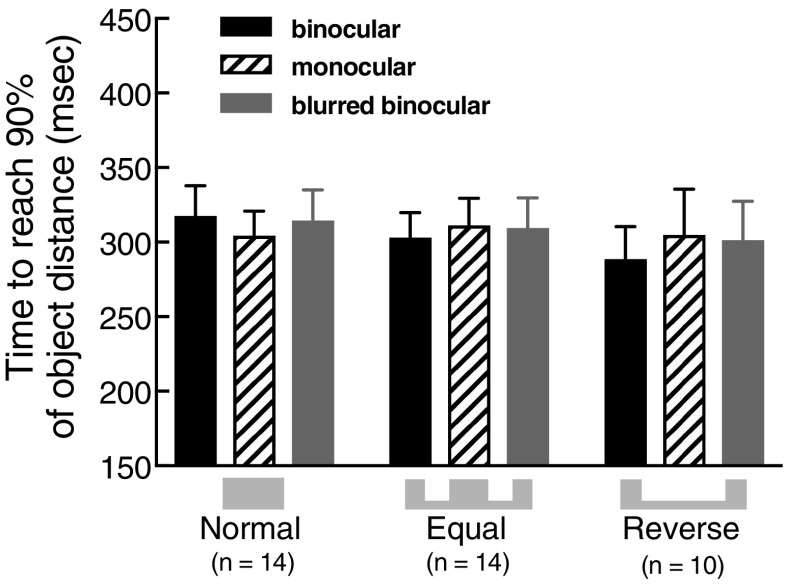
Movement time results. Average time required for the hand position (average of finger and thumb markers) to reach 90% of the target object distance in each probability condition and viewing condition. Error bars denote ± 1 SEM (between subjects)

Although the pattern in our grip aperture results appears straightforward, caution must be exercised in interpreting these data in isolation. Grip apertures can be ‘traded off’ against movement speed, with faster movements associated with larger grasp opening (in itself presumably a form of margin-for-error response) and vice versa (Wing et al. 1986). Such interactions could alter the conclusions that can be drawn from our data. For example, in our reverse condition, the observed reduction in grip apertures could, in-principle, result from moving more slowly with increased uncertainty, rather than sensitivity to the (altered) probability landscape per se. We examined whether overall movement speed and grip aperture were traded off in this manner by analysing the effects of viewing condition on movement times within each probability condition. We needed to measure only the planned responses to increased visual uncertainty, excluding the time after object contact in which the hand is under haptic feedback control. We therefore characterised movement time on each trial as the time elapsed between the hand releasing the start button and reaching 90% of the object’s distance, measured straight ahead along the table surface (here, the *z*-dimension). We calculated hand position by averaging *z*-position of the finger and thumb markers for each recorded frame. Figure 4 plots the overall mean of these data in each condition, collapsed across object size and distance. It can be seen that movement time was unaffected by visual uncertainty in all three probability-landscape conditions. One-way repeated-measures ANOVAs showed that movement times were not significantly affected by visual uncertainty in either the normal (*F*(2,13) = 1.08; *p* = 0.35), equal (*F*(2,13) = 1.37; *p* = 0.27), or reverse (*F*(2,9) = 1.77; *p* = 0.20) probability-landscape conditions. On this basis, we conclude that it is meaningful to interpret the grip aperture effects at face value.

## Discussion

In this experiment, we explored the intuitive idea that hand pre-shaping in grasping movements is programmed so as to actively control the probability of errors in face of (visual) uncertainty. We argued that such behaviour requires not only sensitivity to the amount of visual uncertainty (Schlicht and Schrater 2007a), but also knowledge of how different possible movements affect the probability of error for a given task—its ‘probability landscape’—if an appropriate movement is to be programmed. To test this, we examined three different grasping tasks, each with very different probability landscapes, for which the appropriate responses to increased uncertainty were qualitatively different. The pattern of grip aperture changes with increased uncertainty closely matched the predictions for a system acting to mitigate the otherwise increased risk of error. Specifically, the same manipulations of visual uncertainty resulted in different, yet appropriate, grip aperture responses in the three conditions. This finding demonstrates that the grasping system is sensitive to the probability landscape associated with different tasks, strengthening the evidence that grasp pre-shaping includes programming of a true margin-for-error component.

We used two different visual manipulations (blurring both eyes’ images, and removing binocular information by covering one eye), because we reasoned this might provide converging evidence about the effects of increasing visual uncertainty. This is what we found. In the normal and reverse conditions, both manipulations produced significant grip aperture changes in the predicted directions. Moreover, the ordering of effects was the same in the two probability conditions: blurring of vision produced systematically larger effects than removing binocular information in both cases. This consistency makes it likely that our effects were due to changes in visual uncertainty per se (with blurring causing a higher level of visual uncertainty than removal of binocular information), rather than other, uncontrolled variables. It has previously been suggested, for instance, that grip aperture changes when binocular information is removed may be driven by biased estimates of object size from monocular depth cues (Servos et al. 1992; but see; Keefe et al. 2011). The fact that the same uncertainty manipulations caused increased and decreased grip apertures (or no change), as appropriate to different conditions, strongly suggests that visual uncertainty was the underlying factor in grip aperture changes in our study.

Figure 3 suggests that visual uncertainty had an overall smaller effect on grip apertures in the reverse condition, compared to the normal condition. It may not be meaningful to directly compare these effects, given the different tasks and starting grip apertures. It seems likely, however, that the relatively unusual nature of the reverse-grasping task may have played a role. The normal task was essentially an everyday movement, and so subjects could know the probability landscape at the outset. In contrast, the reverse task was relatively novel, and so subjects may have been to some extent learning the task and associated probability landscape during the experiment. As discussed in the “Results”, if the grasping system has less knowledge of how to pre-shape the hand to match object size, it makes sense that this would also compromise the ‘additional’ step of computing a margin for error around that size. Similarly, poorer knowledge of the probability landscape per se would affect the ability to calculate the margin for error appropriately. The relatively low scaling of minimum grip apertures with object size in the reverse condition (and the fact that a substantial proportion of our subjects showed no reliable scaling at all) is consistent with the task being less well learned. Thus, we speculate that the smaller effect of uncertainty in the reverse conditions was due to this relative lack of learning of the task, and associated probability landscape, compared to normal grasping (see also the discussion of error rates, below), rather than a fundamental difference in how uncertainty was managed in the two conditions.

We have assumed that the goal of a margin-for-error mechanism is to control the probability of errors to some criterion level across changes in visual uncertainty. Our grip aperture data are qualitatively consistent with such a mechanism, but do not indicate whether error rate control was actually achieved. We consider this here. Specifically, we examine whether error rates remained constant across changes in visual uncertainty in the normal and reverse conditions, in which grip apertures could be adjusted to control them. No such mitigating adjustment was possible in the equal condition, so error rate should increase with increased uncertainty. Note, our experiment was not designed to provide a definitive test of this aspect of margin-for-error control, and so this analysis is necessarily speculative.

**Fig. 5:**
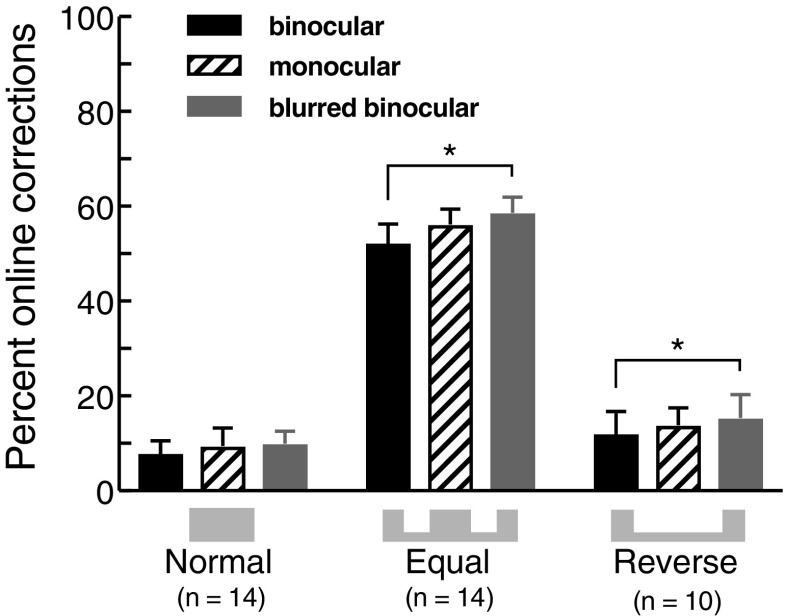
Online corrections to movements. Overall mean percentage of trials containing online corrections or secondary movements in each probability and viewing condition (see main text for definition). Error bars denote ± 1 SEM (between subjects)

Complete failure to grasp objects was vanishingly rare because haptic feedback initiates secondary movements, or online corrections, when objects are not grasped cleanly at the first try (Marotta and Goodale 1998). We defined error rate as the percentage of trials on which such online corrections occurred, defined as trials containing non-monotonic bumps in grip aperture exceeding 5 mm during the closure phase. Figure 5 plots the overall mean percentage of trials containing online corrections in each condition. Assuming that factors affecting the criterion error level—energetic costs of movements (Shadmehr et al. 2016), and precision requirements, for instance—were constant within each probability condition, perfect error control would be evident as constant error rate across changes in visual uncertainty. For the normal condition, error rates were low overall, and did not change appreciably with increased uncertainty. We tested for statistically significant effects using *t* tests, not corrected for multiple comparisons, to give the highest probability of finding departures from the predicted constant error rate (i.e., the highest likelihood of rejecting the statistical null hypothesis). Tests of both binocular vs. monocular viewing (*t*(13) = 1.34, *p* = 0.203) and binocular vs. blurred viewing (*t*(13) = 1.24, *p* = 0.237) were non-significant, consistent with effective control over error rates in normal grasping. Error rates in the reverse condition appear to increase somewhat with visual uncertainty (Fig. 5). Using the same statistical tests as above, the binocular vs. monocular comparison was not significant (*t*(9) = 1.01, *p* = 0.338), but the binocular vs. blurred comparison was significant (*t*(9) = 2.52; *p* = 0.033). Although small, this effect is nonetheless inconsistent with perfect control of error rates, despite the qualitatively appropriate reductions in grip aperture we observed. We speculate that this, too, points to the task and/or probability landscape for this condition being learned less well than in normal grasping, compromising the calculation of an optimal margin for error. In the equal condition grip aperture adjustments—larger or smaller—could not provide effective control over the (increased) probability of errors. Our analysis of movement time (Fig. 4) indicates that our subjects did not instead adopt the alternative error-mitigation strategy of moving more slowly with increased visual uncertainty (Wing et al. 1986) (perhaps this would not have been effective, given the high precision requirement of the equal task, and the fact that one advantage of moving more slowly is increased time to process visual feedback, which was unavailable here?). Logically, then, error rates should rise with increased uncertainty, and this appears to be the case. Figure 5 suggests that error rates were not only substantially higher overall for the equal task than the other two tasks (unsurprisingly, given the increased precision requirement), but also increased with increased visual uncertainty. Using the same *t* test comparisons as previously, the binocular vs. monocular difference was not significant (*t*(13) = 1.34, *p* = 0.204), but the error rate in the blurred condition was significantly higher than in the binocular condition (*t*(13) = 2.59; *p* = 0.023). Overall, this analysis is consistent with the idea that the adjustments to grip apertures we observed resulted in largely effective control of error rates, where possible. Further studies are required to test this idea definitively, however.

Our study used task-dependent changes in the probability landscape. These were relatively naturalistic, but also confounded changes in the probability landscape with the nature of the required movement. It would be informative in future to manipulate the probability landscape while holding the task constant (for instance in a virtual visual-haptic environment, where success at the task could be controlled independently of the actual movement produced, and probability landscapes could be specified, quantitatively, by the experimenter). Such an approach would allow investigation of whether probability landscapes (and visual uncertainty) are managed in a quantitatively correct manner by the grasping system (as opposed to the qualitative approach we employed), and may provide a useful window on how new probability landscapes are learned.

The margin-for-error idea presents a challenging problem for selecting the appropriate movement in a given situation, because it implies the system must search the space of all possible movements, considering the probability of error in each case. This problem is not specific to considering the probability of errors, however, but applies to movement selection in general, where there is a potentially boundless number of ways to perform a given action, but a single movement must ultimately be chosen and executed. Recent models have proposed that the specification of movements (determining their precise spatiotemporal parameters) and movement selection comprise a single process, carried out by a common mechanism, involving competition between different possible movements (e.g. Cisek 2007). We suggest that factoring in the probability of errors (along with other variables such as the value of outcomes; e.g., Christopoulos et al. 2015) must be an integral part of such a process.

It should also be emphasised that the idea of a margin-for-error in grasping, as discussed here, is not tied to our experiment parameters of object size and visual uncertainty, or to particular models of grasp control. Our manipulation of visual uncertainty does not distinguish between uncertainty about object size and position, because both properties derive from the same signal (the precision with which the object’s surfaces can be localised). These properties are dissociable, however, such as when memory for familiar objects provides precise size, but not location, information. Studies of memory-guided grasping suggest that grip apertures are also adjusted in a margin-for-error-like manner in response to uncertainty in object position per se (Hesse et al. 2016). Moreover, although our study is framed in terms of the relationship between hand opening and object size, this does not imply that grip aperture is necessarily an explicitly controlled component of grasping movements (Jeannerod 1984, 1988). Other possibilities exist, including that grip apertures are merely an emergent property of a control process that operates on the separate digits (Smeets and Brenner 1999; see also; Volcic and Domini 2016). In our view, the concept of a margin for error applies regardless of the specific control mode (although implementation would differ), because the individual digits still need to approach the object in a way that reflects uncertainty about the position of its surfaces if the probability of errors is to be controlled.

The finding that the margin for error in grasping is configured appropriately across changes in task adds to the evidence that the brain not only estimates properties of objects to be grasped, but also encodes the amount of uncertainty in those estimates, and manages it appropriately (Schlicht and Schrater 2007a; Christopolous and; Schrater 2009; Takemura et al. 2015). Theories of statistically optimal sensory integration that have emerged in the past 2 decades emphasise the importance of considering the noise/uncertainty inherent in sensory processing. Specifically, these theories specify how sensory signals would ideally be combined in a manner that reflects their relative informativeness in a given situation (Ernst and Banks 2002; Ghahramani et al. 1997; Jacobs 1999; Oruç et al. 2003). Key advantages of such a mechanism (as opposed to being hard-wired to rely on a particular sensory signal) are that object properties can be estimated robustly across substantial variations in signal informativeness that occur in natural viewing (Gepshtein and Banks 2003; Knill and Saunders 2003; Hillis et al. 2004; Greenwald and Knill 2009; Keefe et al. 2011), and that it allows the brain to estimate object properties with the highest possible precision (least possible uncertainty) given the available information (Yuille and Bülthoff 1996; Clark and Yuille 1990; Landy et al. 1995; Oruç et al. 2003; Knill and Pouget 2004). Human perceptual performance has been shown to closely approximate this statistically optimal ideal in domains relevant to grasp control, including visual perception of surface orientation (Knill and Saunders 2003; Hillis et al. 2004) and visual-haptic size estimation (e.g. Ernst and Banks 2002; Gepshtein and Banks 2003). Yet the extent to which perceptual optimality confers functional advantages in everyday situations, or has consequences for conscious perception remains unclear (do we perceive differences in perceptual uncertainty, for instance?) (Vishwanath and Hibbard 2013). The benefits of optimally precise estimates for motor control tasks such as grasping are more readily apparent, however. For instance, they can be used to programme a smaller margin for error in grasping, presumably helping to minimise energetic costs associated with a movement, while still controlling the probability of success (and therefore the value associated with the movement outcome).

In summary, we have shown that grasp pre-shaping responds to increased visual uncertainty in a manner that closely resembles a margin for error. Specifically, we found that grasping is sensitive to changes in the required response to increased uncertainty. That is, movements were adjusted appropriately across substantial changes in the relationship between different possible movements and the probability of errors (the probability landscape), such that the same manipulations of visual uncertainty resulted in very different changes in grasp pre-shaping (wider, smaller, or unchanged hand opening), as appropriate to controlling the probability of errors. Our results support the intuitive idea that grasp programming includes a true margin for error, which is flexibly specified in response to different circumstances, exerting active control over the probability of errors. As such, our results also further underline the idea that understanding how noise/uncertainty is managed is a critical aspect of understanding sensorimotor processing more generally.
